# The triglyceride-glucose index in chronic kidney disease: a narrative review

**DOI:** 10.3389/fnut.2026.1758394

**Published:** 2026-03-09

**Authors:** Yaqing Wang, Yuqing Wang, Yiran Ge, Xiaojie He, Xiaodong Li

**Affiliations:** 1 Graduate School of Chengde Medical University, Chengde, Hebei, China; 2 Graduate School of Hebei Medical University, Shijiazhuang, Hebei, China; 3 Department of Nephrology, Baoding No. 1 Central Hospital of Hebei Medical University, Baoding, Hebei, China

**Keywords:** chronic kidney disease, insulin resistance, pathogenesis, prognosis, TyG index

## Abstract

Chronic kidney disease (CKD), defined by the progressive and irreversible deterioration of renal structure and function stemming from diverse etiological factors, poses a formidable and escalating public health burden on a global scale. Concurrently, insulin resistance (IR), a pathophysiological state characterized by diminished cellular responsiveness to insulin, presents a complex and multifactorial metabolic derangement. The triglyceride-glucose (TyG) index has emerged as a robust, accessible, and reliable surrogate marker for quantifying this IR status. A growing body of evidence underscores a profound and intricate interconnection between the IR state and the pathogenesis, initiation, and advancement of CKD. Delineating this relationship carries significant consequential value for the enhancement of early identification, refined risk stratification, and improved therapeutic management and preventive strategies for CKD. This article conducts a comprehensive narrative review and critical analysis of contemporary scientific investigations pertaining to the applicability and prognostic utility of the TyG index in forecasting, diagnosing, and evaluating the progression and clinical outcomes of CKD. Through a synthesis of pertinent literature, this review further elucidates the potential mechanistic pathways linking the TyG index to CKD pathogenesis, encompassing its influence on metabolic homeostasis, the exacerbation of insulin resistance, and the propagation of chronic inflammatory processes. The overarching objective is to contribute novel evidentiary support for predicting the trajectory of CKD, its associated complications, and long-term prognoses, thereby proposing innovative avenues for pioneering clinical diagnostics and preemptive interventional measures.

## 1 Introduction

Chronic kidney disease (CKD) is a complex multisystem disorder frequently characterized by non-specific early-stage symptoms, often resulting in diagnosis at advanced stages. This not only increases therapeutic complexity and healthcare costs but also substantially diminishes patients’ quality of life. Progression to end-stage renal disease (ESRD) necessitates life-sustaining dialysis or kidney transplantation, imposing substantial burdens on both society and families. According to the latest Global Burden of Disease (GBD) study published in The Lancet, approximately 788 million adults worldwide were affected by CKD in 2023, corresponding to a prevalence of 14.2%—a marked increase from 378 million in 1990 (1). In 2019, CKD was responsible for 1.4 million deaths globally, ranking as the eighth leading cause of non-communicable disease mortality, with associated health and economic burdens rising rapidly (2–6).

Studies indicate that varying degrees of insulin resistance (IR) are prevalent among CKD patients (7). IR exacerbates renal impairment through multiple pathophysiological mechanisms and plays a critical role in the development and progression of kidney disease [8]. As a core component of metabolic syndrome, IR promotes the initiation and progression of CKD via pathways including inflammation, oxidative stress and endothelial dysfunction. The triglyceride-glucose (TyG) index, which integrates two key metabolic parameters—triglycerides and fasting glucose—reflects the lipid and carbohydrate metabolic dysregulation commonly observed in renal impairment (8, 9). Its straightforward calculation and low cost render it highly attractive for clinical application across diverse healthcare settings.

Emerging evidence demonstrates that the TyG index not only correlates with established markers of IR but also holds significant value in predicting CKD onset, progression and related complications (8, 10, 11). Numerous studies consistently confirm that elevated TyG index levels are strongly associated with decreased estimated glomerular filtration rate (eGFR) and increased urinary albumin (12–15).

This review systematically evaluates current evidence regarding the clinical utility of the TyG index in CKD, focusing on: its predictive performance for CKD incidence and progression; prognostic implications for complications; potential mechanistic pathways; and clinical implications. By synthesizing recent epidemiological and clinical research findings, this article aims to objectively assess the potential value of this biomarker in CKD management and to propose future research directions.

## 2 Methodology

The review was conducted following a systematic literature search to ensure a comprehensive and transparent synthesis of evidence. We searched the PubMed, Web of Science, and Scopus databases for relevant articles published from January 2001 up to December 2025. The search strategy employed a combination of keywords and MeSH terms, including: “Triglyceride-glucose index” OR “TyG index” AND “chronic kidney disease” OR “CKD” OR “renal insufficiency” OR “end-stage renal disease” OR “kidney function” OR “albuminuria.” We also manually screened the reference lists of included articles and relevant reviews to identify additional studies.

Studies were included if they were original research articles or systematic reviews/meta-analyses published in peer-reviewed journals, written in English, and investigating the association between the TyG index and CKD incidence, progression, or related complications. We excluded editorials, conference abstracts, case reports, and studies not available in full text. The selection of studies for inclusion in this narrative review was based on their relevance to the predefined thematic areas: epidemiological evidence, pathophysiological mechanisms, and clinical utility of the TyG index in CKD.

## 3 The TyG index and IR

IR refers to a reduced responsiveness of insulin-targeting organs or tissues—primarily muscle, adipose tissue, and liver—to elevated insulin levels, often leading to impaired glucose metabolism. It is widely recognized as a key pathogenic driver of numerous metabolic disorders, including type 2 diabetes mellitus (T2DM), atherosclerosis, and non-alcoholic fatty liver disease. Furthermore, emerging evidence suggests potential links between IR and certain cancers, neurodegenerative diseases, psychiatric conditions, and frailty (16–18). The hyperinsulinemic–euglycemic clamp is the gold standard method for quantifying tissue sensitivity to insulin. However, its complexity, time-consuming nature, and high cost limit its application to small-scale scientific studies (19, 20). In larger epidemiological and clinical research, the homeostatic model assessment of IR (HOMA-IR) is more widely used (21). HOMA-IR estimates the degree of IR from fasting plasma insulin and glucose concentrations, and its results correlate well with those obtained via the clamp technique (22). Nevertheless, its reliance on accurate fasting insulin measurement restricts its utility in primary care settings and less developed regions.

The TyG index, a biochemical marker gaining increasing attention in both clinical and research settings, is derived from fasting triglyceride and glucose levels. It was first introduced by Simental-Mendía et al. in 2008 in a cross-sectional study of healthy individuals, using the formula: TyG index = ln[(fasting triglycerides (mg/dL) × fasting glucose (mg/dL)) / 2] (23). To ensure accuracy, measurements should be taken after a fast of 8–12 h. In 2010, Guerrero-Romero et al. demonstrated, through comparative analysis of individuals with varying body weights and glycemic statuses, that the TyG index had high sensitivity (96.5%) and specificity (85.0%) for identifying IR compared to the clamp technique (24). This finding highlighted its effectiveness in detecting diminished insulin sensitivity. Subsequently, scientific interest in the TyG index has grown substantially. Numerous large-scale clinical studies have now established it as a reliable surrogate marker for assessing IR in at-risk populations. Its technical simplicity and low cost make it an attractive and practical alternative for widespread clinical use.

## 4 The relationship between the TyG index and CKD

### 4.1 Epidemiological evidence for the association between the TyG index and CKD

#### 4.1.1 Evidence from cross-sectional studies

Cross-sectional studies provide extensive preliminary epidemiological evidence linking the TyG index to CKD. This association has been observed across diverse populations with varying geography, ethnicity, and clinical characteristics. In the general population, a large cross-sectional analysis of NHANES data revealed a significant positive correlation between the TyG index and CKD prevalence among US adults (OR = 4.03; 95% CI: 1.81–8.96). This study also identified a unique J-shaped relationship in adults aged 41–60 years, where CKD risk increased sharply once the TyG index exceeded a certain threshold. Moreover, the association between the TyG index and albuminuria was even more pronounced (OR = 6.11; 95% CI: 2.64–14.14), suggesting a potential strong link with early kidney damage markers (25). Data from the China Health and Retirement Longitudinal Study (CHARLS), another large cross-sectional study, showed that a higher TyG index significantly increased the odds of CKD. When participants were stratified by TyG index quartiles, the highest quartile (Q4) had a significantly greater risk of CKD than the lowest (Q1), a trend that persisted after adjusting for confounders such as residence, history of dyslipidemia, diabetes, smoking, alcohol consumption, body mass index (BMI), and blood pressure (26).

The association is particularly pronounced in certain disease populations. A 2024 cross-sectional analysis indicated a positive correlation between the TyG index and CKD prevalence in individuals with metabolic dysfunction-associated fatty liver disease (MAFLD), particularly among those under 60 years (27). A cross-sectional study by Chen et al. in Eastern China found that among patients withT2DM, the TyG-BRI index (which combines the TyG index with the Body Roundness Index) was positively associated with CKD risk, with the Q4 group having 1.57 times the odds of CKD compared to Q1 (OR = 1.57; 95% CI: 1.10–2.25) (28). Another study reported significantly elevated TyG indices in patients with dyslipidemia and hyperuricemia—both established risk factors for CKD (29). Collectively, these findings suggest that the relationship between the TyG index and CKD may be stronger in individuals with pre-existing metabolic disturbances.

#### 4.1.2 Evidence from cohort studies

Prospective cohort studies, with their long-term follow-up, provide higher-level evidence regarding the potential causal association and predictive capacity of the TyG index for incident CKD. A prospective cohort study of 3,439 individuals aged 40–75 demonstrated that a high TyG index was a risk factor for the development of abnormal eGFR and for the onset and progression of CKD. Participants with a TyG index > 9.20 had a 1.815-fold higher risk of developing CKD compared to those with a TyG index ≤ 8.47 (30). A retrospective cohort study by Hou Q et al., which followed 4,921 healthy individuals for up to 8 years, modeled longitudinal TyG index trajectories into three groups: high-stable, moderate-stable, and low-stable. Results indicated that participants in the high-stable trajectory group had a significantly increased risk of incident CKD compared to those in the low-stable group (HR = 2.399; 95% CI: 1.167–4.935). This suggests that tracking the dynamic change of the TyG index over time may more sensitively reflect long-term metabolic burden and improve the prediction accuracy for CKD risk compared to a single baseline measurement (31).

The strength of association varies across population subgroups. A study of Finnish men (baseline age 42–61 years with normal renal function) with a median follow-up of 17.5 years found that after adjusting for traditional risk factors, each 1-unit increase in the TyG index was associated with a 59% higher risk of incident CKD (HR = 1.59; 95% CI: 1.24–2.05). Furthermore, after additional correction for regression dilution bias due to within-individual variation, the association between the TyG index (based on repeated measurements) and CKD risk was even stronger, implying that reliance on a single baseline measurement may substantially underestimate the true association (32). In MAFLD patients, each 1-unit increment in the TyG index was associated with a 19% higher risk of CKD development (HR = 1.19; 95% CI: 1.09–1.29), with this association being more marked in patients under 60 years (27). For diabetic patients, research by Rodzoń-Norwicz et al. highlighted the importance of phenotypic analysis, revealing distinct metabolic and molecular marker profiles across different diabetic kidney disease phenotypes, which might explain the heterogeneity in the TyG index’s predictive power among individuals (33).

#### 4.1.3 Discussion on strength and consistency of evidence

Synthesizing evidence from cross-sectional and cohort studies, the majority support a positive association between the TyG index and CKD (25–36). However, some heterogeneity exists across studies. For instance, one study found no significant difference in CKD risk across TyG quartiles when using only a single baseline measurement for grouping (31). Such inconsistencies may arise from several factors: (1) Population differences: Variations in ethnicity, age, and underlying comorbidities (e.g., healthy individuals vs. those with T2DM, MAFLD, or hypertension) likely influence the degree of IR and the mechanisms of renal injury. (2) Scope of covariate adjustment: Differences in the set of confounders adjusted for in statistical models (e.g., inclusion or omission of inflammatory markers, specific medications) may lead to residual confounding. (3) Measurement and modeling of the TyG index: Studies using a single baseline measurement versus those employing repeated measures or trajectory modeling capture different aspects of exposure; the latter better reflects long-term metabolic load.

Notwithstanding this heterogeneity, the overall direction of findings from most studies is consistent: a higher TyG index is a risk factor for the development and progression of CKD. This association appears more robust and clearly defined in high-risk populations, such as those with MAFLD, diabetes, or individuals maintaining a persistently high TyG index trajectory. To succinctly summarize the characteristics and key findings of the major cross-sectional and prospective cohort studies discussed, they are presented in the Table 1.

**Table 1:** Summary of characteristics and key findings from major epidemiological studies on the association between the TyG index and CKD.

Study type	Author/Ref. (Year)	Study population	Sample size	Follow-up duration	Key findings
Cross-sectional analyses	Li et al. (25)	American adults (NHANES)	18,078	\	TyG index positively associated with CKD (OR = 4.03); J-shaped relationship in 41–60 years group.
Cross-sectional analyses	Chen et al. (28)	Chinese patients with T2DM	1,756	\	TyG-BRI Q4 group: 1.57 × higher CKD risk vs. Q1.
Cross-sectional analyses	Xiao Ren et al. (35).	Chinese adults ≥45 years old	10,498	\	TyG Q4 vs. Q1: significantly increased CKD prevalence risk.
Prospective cohort studies	Kunutsor et al. (32)	Finnish male participants, aged 42–61 years	2,362	Median: 17.5 years	Per 1-unit TyG increase: CKD HR = 1.59; association strengthened after regression dilution bias adjustment.
Retrospective cohort studies	Hou et al. (31)	Health check-up population in china	4,921	8 years	High TyG trajectory vs. low-stable: CKD HR = 2.399.
Retrospective cohort studies	Wei et al. (27)	Adult MAFLD patients	11,860	Median: 10.02 years	Per 1-unit TyG increase: CKD HR = 1.19; association stronger in participants <60 years.

TyG, Triglyceride-Glucose Index; CKD, Chronic Kidney Disease; T2DM, Type 2 Diabetes Mellitus; MAFLD, Metabolic Dysfunction-Associated Fatty Liver Disease; NHANES, National Health and Nutrition Examination Survey; BRI, Body Roundness Index; OR, Odds Ratio; HR, Hazard Ratio.

### 4.2 Prognostic and clinical utility of the TyG index in CKD progression, complications, and outcomes

The clinical application of the TyG index in CKD extends beyond early identification and risk prediction, demonstrating significant potential for monitoring disease progression, assessing complication risks, and determining prognosis.

#### 4.2.1 Predicting CKD progression

Evidence indicates a close association between the TyG index and the decline of renal function in CKD patients. A higher TyG index is linked to an increased risk of progression to ESRD (37). Specifically, in patients with CKD stages 3–5, the TyG index shows significant correlations with renal function parameters, including the eGFR, as well as with body composition metrics, suggesting its utility as a valuable tool for assessing the risk of CKD progression (38).

#### 4.2.2 Assessing cardiovascular complications

CKD patients face a substantially elevated risk of major adverse cardiovascular events (MACE), with cardiovascular mortality rising as renal function declines. A study involving 1,142 non-diabetic CKD patients and 460 controls revealed significantly higher TyG index levels in the CKD group, which correlated with MACE incidence. Both univariate and multivariate analyses confirmed the TyG index as an independent predictor of MACE, potentially aiding risk stratification and guiding primary cardiovascular disease prevention strategies in this patient population (39).

The predictive value of the TyG index remains significant in patients receiving renal replacement therapy. A prospective cohort study by Yan et al. of 3,054 incident peritoneal dialysis patients found that the baseline TyG index was significantly associated with cardiovascular mortality after adjusting for potential confounders. Patients in the Q4 had nearly triple the risk of cardiovascular death compared to those in the Q1 (40). Even in the high-risk population of ESRD patients with concomitant coronary artery disease, the TyG index retained its prognostic value. Research by Xie Ed et al. similarly showed that patients in the highest TyG index tertile had a 63% increased risk of MACE compared to those in the lowest tertile. For each 1-unit increase in the TyG index, MACE risk rose by 37%, and the index provided incremental predictive value beyond traditional risk scores (41).

#### 4.2.3 Evaluating non-cardiovascular complications

The TyG index is also significantly associated with non-cardiovascular complications in CKD. Studies indicate a positive correlation between the TyG index and the prevalence of sarcopenia in CKD patients. In a US cohort, the highest TyG quartile was associated with a 4.01-fold higher risk of sarcopenia compared to the lowest quartile; this risk was 3.25-fold in a Chinese cohort. Notably, this association was particularly pronounced in patients without coexisting diabetes or metabolic syndrome, suggesting that underlying metabolic comorbidities may exert an effect-modifying influence (42). Furthermore, a study of 208 peritoneal dialysis patients demonstrated that the baseline TyG index was an independent risk factor for the first episode of peritoneal dialysis-related peritonitis. Patients with a higher TyG index faced a greater infection risk, and the index positively correlated with inflammatory parameters (43).

#### 4.2.4 Prognostic prediction across different CKD populations

The TyG index demonstrates robust prognostic ability across various CKD subpopulations. A study by Su C et al., which enrolled 717 CKD patients with advanced chronic heart failure, identified a high TyG index as a risk factor for both MACE and all-cause mortality. The high-TyG group exhibited a 440.2% increased risk of MACE and a 406.2% increased risk of all-cause mortality, establishing the TyG index as an independent prognostic predictor in this cohort (44). In non-dialysis dependent CKD stages 3–5, Kaplan–Meier analysis indicated that patients with higher TyG indices experienced higher rates of adverse clinical outcomes. The optimal TyG index cut-off values for predicting heart failure rehospitalisation and cardiovascular death were 1.343 and 1.436, respectively. Higher TyG index levels correlated with worse cardiovascular outcomes, and the index was an independent predictor of heart failure (45). A retrospective cohort analysis by Zhang Y et al. of 368 maintenance hemodialysis patients found the TyG index to be an independent predictor of all-cause and cardiovascular mortality. Patients with a high TyG index had 1.790 and 1.735 times the risk of all-cause and cardiovascular death, respectively, compared to those with a low index (46). Notably, a U-shaped association was observed in the dialysis subgroup: both extremely low and high TyG index values were associated with increased mortality risk (47). This non-linear relationship underscores the complexity of metabolic dysregulation in ESRD and suggests the potential existence of an optimal TyG index range for clinical outcomes.

In summary, the TyG index, as a novel biomarker of IR, shows significant associations with the incidence, progression, and prognosis of CKD. Existing evidence supports its role as an independent indicator for assessing CKD risk and highlights its broad potential for clinical application. However, future large-scale, multicenter studies with long-term follow-up are needed to further validate its accuracy and applicability in renal diseases and to elucidate its specific performance across different patient populations. Building upon this evidence, the TyG index holds promise as an important tool in the clinical management of CKD, potentially aiding clinicians in better assessing renal risk, formulating personalized treatment strategies, and improving patients’ quality of life.

It is important to note that the predictive value and clinical utility of the TyG index may vary across different stages of CKD. The majority of evidence, particularly regarding incident CKD, is derived from populations with preserved or mildly decreased renal function. In CKD stages 4–5 and in patients on renal replacement therapy, the TyG index continues to demonstrate strong prognostic value, especially for cardiovascular outcomes and mortality (41, 46). However, the interpretation of the index in advanced stages is complicated by factors like protein-energy wasting and altered lipid metabolism, which may weaken its association with IR or even create non-linear relationships (47). Data specifically on the utility of the TyG index for monitoring progression from early to intermediate stages of CKD remain scarce, representing a critical area for future investigation.

### 4.3 Potential mechanisms linking the TyG index to CKD

As previously discussed, the TyG index is a well-validated surrogate marker for IR and is closely associated with the development and progression of CKD (48, 49). The underlying mechanisms involve multiple interconnected pathways, primarily including IR and related metabolic disturbances, inflammation and oxidative stress, endothelial dysfunction, activation of the renin-angiotensin-aldosterone system (RAAS), and other cell-specific injury pathways. Elucidating these mechanisms is crucial for understanding the specific role of the TyG index in CKD progression and provides a theoretical basis for developing novel intervention strategies (50–54). It is important to emphasize that a multifactorial, bidirectional interaction exists between kidney injury and IR: CKD itself can induce or exacerbate IR, creating a positive feedback loop that further drives disease progression (44).

#### 4.3.1 IR and metabolic syndrome

IR constitutes the core pathophysiological basis linking the TyG index to the onset and progression of CKD. In the state of IR, reduced tissue sensitivity to insulin leads to compensatory hyperinsulinemia. This metabolic disturbance contributes to renal injury through multiple direct and indirect mechanisms. Direct Pathways: Aberrant insulin signal transduction is a key event. This is primarily characterized by increased serine phosphorylation of insulin receptor substrate (IRS) 1/2, which subsequently inhibits the normal activation of the PI3K/Akt signaling pathway (55). Notably, this pathway is physiologically crucial for its anti-apoptotic effects and for maintaining the functional integrity of glomerular podocytes; its impairment directly disrupts renal cellular homeostasis (56). Indirect Pathways: The lipotoxic effects triggered by IR are particularly prominent. Elevated circulating free fatty acid levels lead to increased renal uptake, exceeding the tissue’s fatty acid oxidation capacity and resulting in abnormal lipid deposition within the kidneys (57). This lipid accumulation can induce apoptosis and a pro-fibrotic phenotypic shift in renal tubular epithelial cells and podocytes, characterized by triglyceride deposition and the generation of toxic lipid metabolites like ceramides. Furthermore, IR is often accompanied by the accumulation of advanced glycation end products (AGEs). AGEs bind to their receptor (RAGE), activating downstream inflammatory pathways such as NF-κB and promoting the synthesis and deposition of extracellular matrix proteins like collagen I and III, directly contributing to glomerulosclerosis and tubulointerstitial fibrosis (58–60).

Notably, the components of metabolic syndrome (e.g., obesity, hypertension, dyslipidemia) can act synergistically with the TyG index to accelerate CKD progression. For instance, obesity-related adipose tissue dysfunction releases a plethora of adipokines, characterized by increased leptin and decreased adiponectin, which further exacerbate renal inflammation and fibrosis.

The kidney is the primary site for clearing plasma leptin. As renal function declines, plasma concentrations of this peptide progressively increase (61). Additionally, enhanced leptin secretion from adipose tissue may further elevate circulating leptin levels in kidney disease patients (62). Leptin contributes to insulin resistance through inhibition of fatty acid oxidation and activation of the IRS/PI3K pathway (63). Clinical studies have confirmed positive correlations between leptin levels and HOMA-IR in dialysis patients and across various CKD stages (64–67).

Adiponectin exerts physiological effects by improving endothelial function, inhibiting inflammatory pathways, and enhancing insulin sensitivity (68–71). Multiple studies demonstrate an inverse causal relationship between adiponectin and insulin resistance (72–76).

#### 4.3.2 Inflammation and oxidative stress

An elevated TyG index is closely linked to systemic and renal-localized inflammation and oxidative stress, which are pivotal drivers of CKD progression (77, 78). A cross-sectional study in dialysis patients found a positive correlation between HOMA-IR and inflammation markers. Furthermore, angiotensin II-triggered oxidative stress may play a role in CKD, as the use of angiotensin II blockers in stage 3–4 CKD patients reduces levels of both angiotensin II and inflammatory biomarkers (79). Hyperinsulinemia resulting from IR can activate pro-inflammatory cascades, such as the NLRP3 inflammasome, leading to the production of cytokines like IL-1β and IL-18, which amplify renal inflammation (80–82). Concurrently, mitochondrial dysfunction in the IR state generates excessive reactive oxygen species (ROS), causing direct oxidative damage and activating pro-fibrotic pathways, thereby accelerating renal injury (83, 84).

#### 4.3.3 Endothelial dysfunction and vascular injury

IR induces endothelial dysfunction both systemically and within the kidney through various mechanisms. Notably, the activation of lipoprotein-associated phospholipase A2 (Lp-PLA2) and its product lysophosphatidylcholine (LPC) plays a novel and critical role in endothelial injury. Notably, the activation of the Lp-PLA2/ LPC axis in IR states impairs endothelial nitric oxide synthase (eNOS) function, reducing nitric oxide (NO) bioavailability and promoting endothelial dysfunction (52, 85, 86). Reduced NO bioavailability diminishes vasodilatory capacity and enhances vascular responsiveness to vasoconstrictors like angiotensin II. This can lead to abnormal constriction of the glomerular afferent arteriole, causing intraglomerular hypertension and hyperfiltration, which, over time, ultimately damages the glomerular filtration barrier (87, 88).

#### 4.3.4 Other potential mechanisms

IR can lead to systemic overactivation of the RAAS, with several studies showing elevated levels of renin, angiotensin-converting enzyme, and aldosterone in individuals with IR (89, 90). RAAS and sympathetic nervous system activation impair natriuresis and increase renal tubular sodium reabsorption, leading to volume expansion and hypertension (91). Furthermore, aldosterone potentiates the effects of angiotensin II, contributes to vascular inflammation and remodeling, and stimulates mineralocorticoid receptors in the kidney. It may also promote renal inflammation, fibrosis, podocyte injury, and parietal epithelial cell proliferation (92). Research by Bakris GL et al. suggests that blocking the RAAS pathway may mitigate obesity-related hypertension, thereby improving renal dysfunction (93).

Podocytes, crucial for glomerular barrier integrity, are particularly vulnerable to IR and lipotoxicity, with damage manifesting as foot process effacement, apoptosis, and disrupted insulin signaling, directly initiating and accelerating CKD (94–98).

**Figure 1:**
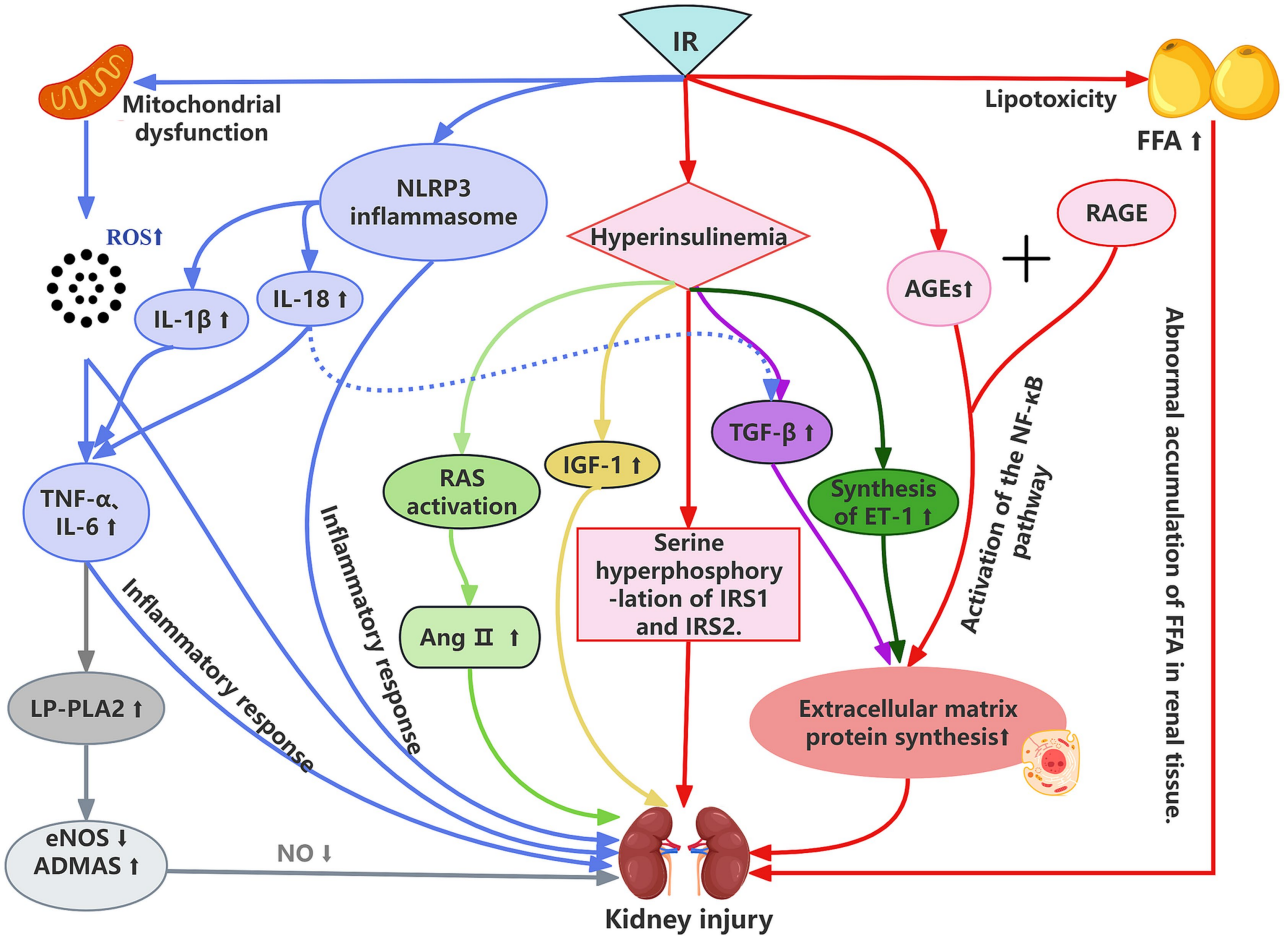
The association mechanism between IR and kidney injury. IR, insulin resistance; IRS1, insulin receptor substrate 1; IRS2, insulin receptor substrate 2; RAS, renin-angiotensin system; IGF-1, insulin-like growth factor-1; GF-β, transforming growth factor-β; ET-1, endothelin-1; AGEs, advanced glycation end products; RAGE, receptor for advanced glycation end products; FFA, free fatty acids; NF-κB, nuclear factor kappa-light-chain-enhancer of activated B cells; NLRP3, NACHT, LRR and PYD domains-containing protein 3; ng II, angiotensin II; IL-8, interleukin-8; IL-1β, interleukin-1β; TNF-α, tumor necrosis factor-α; IL-6, interleukin-6; LP-PLA2, lipoprotein-associated phospholipase A2; eNOS, endothelial nitric oxide synthase; ADAMTS, a disintegrin and metalloproteinase with thrombospondin motifs.

In summary, IR influences the initiation and progression of CKD through a multifaceted network of mechanisms involving metabolic dysregulation, inflammatory cascades, oxidative stress, endothelial dysfunction, and fibrosis, collectively leading to renal structural damage and functional decline. Understanding these intricate pathways is essential for developing early intervention and renal protection strategies targeted at individuals with an elevated TyG index. To provide a holistic overview of these complex, interlinked pathways, we have summarized them in the schematic Figure 1, which delineates how IR contributes to renal damage via multiple avenues.

### 4.4 Clinical potential of the TyG index in CKD risk management

The epidemiological association between the TyG index and CKD, along with its underlying mechanisms, has been extensively documented. However, its true clinical value lies in its potential translation into an effective risk management tool. This section systematically evaluates the clinical potential of the TyG index in the diagnosis, screening, and risk prediction/stratification of CKD. By comparing it with other indicators, we aim to define its advantages and position within CKD management.

#### 4.4.1 Diagnostic and screening value

As a simple surrogate marker of IR, the value of the TyG index for the early identification and screening of CKD has been widely investigated. As shown in Table 2, multiple studies utilizing receiver operating characteristic (ROC) curve analysis have confirmed the good diagnostic performance of the TyG index for CKD (14, 25, 27, 99, 100).

**Table 2:** Summary of key studies on the diagnostic performance of the TyG index for CKD.

Study population	Sample Size	CKD Stages	Optimal Cut-off	Sensitivity (%)	Specificity (%)	AUC (95% CI)	Reference
Chinese non-diabetic population	29,625	eGFR<60	8.53	67.7	54.5	0.644 (0.634–0.655)	Liu et al. (14)
Type 2 diabetic patients	673	eGFR<60 UACR≥30	9.35	67.0	58.5	0.681 (0.641–0.720)	Li et al. (99)
Hypertensive patients	2033	eGFR<60	8.94	70.1	71.3	0.762 (0.731–0.793)	Zhu et al. (100)
US adults (NHANES)	18,078	eGFR<60	8.80	74.5	73.2	0.821 (0.803–0.839)	Li et al. (25)
Fatty liver disease patients	11,860	eGFR<60	8.85	75.2	72.8	0.795 (0.776–0.814)	Wei et al. (27)

eGFR, Estimated Glomerular Filtration Rate, mL/(min·1.73m^2^); UACR, Urine Albumin-to-Creatinine Ratio, mg/g; NHANES, National Health and Nutrition Examination Survey; AUC, Area Under the Curve; CI, Confidence Interval.

Determining the optimal cut-off value is crucial for its screening function. As presented in Table 2, this value varies somewhat across different populations. In general populations, the cut-off for diagnosing CKD typically clusters between 8.5 and 8.8 (14, 25). However, in specific high-risk populations with comorbid metabolic disorders, the optimal cut-off tends to be higher. For instance, it is 9.35 in patients with T2DM and 8.94 in hypertensive patients (99, 100). This variation likely stems from the fact that these disease states are already associated with significant IR and metabolic insult, thereby requiring a higher threshold of IR to identify superimposed renal damage.

#### 4.4.2 Comparison with other indicators

Research by Nabipoorashrafi SA et al. indicated that in patients with type 2 diabetes, the TyG index (AUC: 0.62) demonstrated a stronger association with albuminuria (OR: 1.67) than other IR indicators like HOMA-IR (OR: 1.127) (101). Similarly, a prospective cohort study showed that a higher TyG index was independently associated with the risk of incident CKD, and incorporating the TyG index into traditional CKD risk prediction models significantly improved their predictive capability (26). Studies by Mu X et al. also noted that the TyG index and the TG/HDL-C ratio showed the best performance for predicting diabetic kidney disease (DKD), both outperforming METS-IR (102). Notably, research by Xiangyu Chen et al. suggested that the TyG-BRI index also possessed moderate predictive value for CKD in T2DM patients, with an area under the curve (AUC) of 0.626 (95% CI: 0.597–0.656, *p* < 0.001) (28). These findings imply that combining the TyG index with other parameters may further enhance its diagnostic efficacy.

**Figure 2:**
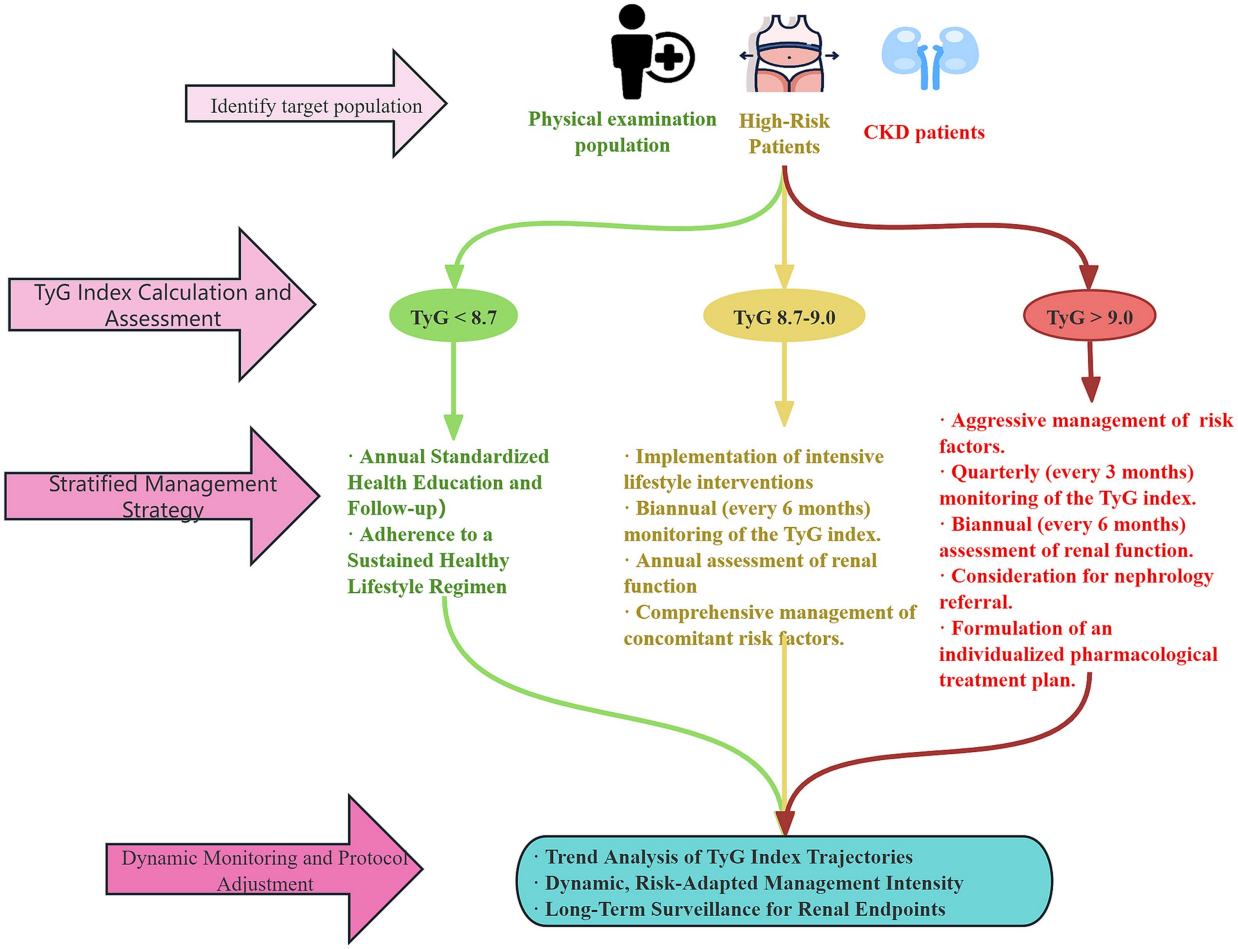
Clinical pathway for TyG index-based CKD risk management. TyG, tyglyceride-glucose index; CKD, chronic kidney disease.

Based on the discussed mechanisms and clinical evidence, we have summarized the clinical advantages of the TyG index and its derived indices (Table 3). Furthermore, integrating existing findings, we propose a preliminary clinical pathway for TyG index-based CKD risk management (Figure 2). This pathway highlights the central role of the TyG index in CKD risk stratification and management, aiming to guide differentiated prevention and management strategies through precise risk assessment.

**Table 3:** Clinical application advantages of the TyG index and its derived indices.

Index	Formula	Clinical application advantages
TyG	ln (Fasting triglycerides [mg/dL] × fasting blood glucose [mg/dL]/2)	Association with metabolic syndrome, diabetes, and cardiovascular diseases
TyG-BMI	TyG × BMI(kg/m^2^)	Prediction of diabetes risk, assessment of metabolic disorders and accumulation of systemic fat in people with a high BMI
TyG-WHtR	TyG-WHtR = TyG × (Waist circumference [cm] / Height [cm])	Early screening for diabetes and assessment of cardiovascular diseases (such as myocardial infarction)
TyG-WC	TyG-WC = TyG × Waist circumference [cm]	Cardiovascular disease risk stratification and CKD risk assessment

TyG, Tyglyceride-Glucose Index; BMI, Body Mass Index; WHtR, Waist-to-Height Ratio; WC, Waist Circumference; CKD, Chronic Kidney Disease.

In summary, the TyG index demonstrates clear potential for clinical application in CKD risk management. Its diagnostic performance is robust, with optimal cut-off values remaining relatively stable across different populations. As an independent predictor, it significantly enhances the stratification capability of existing risk models.

## 5 Conclusion and future perspectives

This review offers a comprehensive assessment of the TyG index and its clinical applicability in CKD. Synthesizing evidence from epidemiological, mechanistic, and clinical studies reveals that this index, as a biomarker closely associated with CKD, holds significant clinical promise. Numerous cross-sectional and cohort studies demonstrate a significant positive correlation between an elevated TyG index and the prevalence, progression, and adverse prognosis of CKD (25–32). For instance, across general populations and specific disease cohorts—such as those with metabolic dysfunction-associated fatty liver disease or T2DM—the index is not only independently associated with CKD incidence but also effectively predicts renal function decline and the occurrence of cardiovascular complications (27, 28, 39–41). The underlying mechanisms are primarily attributable to IR, which promotes renal injury through multiple interconnected pathways, including metabolic dysregulation, activation of inflammatory and oxidative stress responses, and induction of endothelial dysfunction (50–54, 77–79). This emphasizes the index’s central involvement in the pathophysiology of CKD.

At the public health and clinical practice levels, the TyG index also shows considerable potential, particularly for early screening and risk stratification of CKD. Studies confirm its high diagnostic efficacy, with areas under the receiver operating characteristic curve typically ranging from 0.644 to 0.821 (14, 25, 99, 100). Coupled with its simple calculation and low cost, it represents a feasible tool for implementation even in resource-limited settings. When compared to other IR indices, the TyG index demonstrates superior performance in predicting CKD risk and improving the stratification power of risk models (26, 101, 102). Furthermore, its association with CKD complications, such as cardiovascular events and sarcopenia, underscores its value as a comprehensive risk assessment tool (39, 42, 44).

Notwithstanding the substantial body of evidence, the widespread clinical adoption of the TyG index faces several challenges. These include the need for standardization of measurement methods, determination of population-specific cut-off values, and validation of its long-term prognostic utility. Future research should focus on validating its predictive ability across diverse populations through large-scale, multicenter prospective cohorts and exploring its combined use with novel biomarkers. Advancing the standardization and clinical application of the TyG index will be crucial for optimizing CKD prevention and management, enabling earlier interventions and personalized therapies, ultimately reducing the global burden of the disease. Furthermore, the interpretation of the TyG index in patients with advanced CKD, particularly those with uremia, requires caution. The altered metabolism of lipids in uremia, characterized by changes in triglyceride-rich lipoprotein composition and clearance, may not directly reflect the same degree of insulin resistance as in earlier stages, potentially confounding the index’s predictive value.

Future research should focus on validating the predictive ability of the TyG index across diverse populations through large-scale, multicenter prospective cohorts, with a specific emphasis on stratifying patients by CKD stage to delineate its differential utility in early detection versus prognostic assessment in advanced disease. Additionally, exploring its combined use with novel biomarkers is warranted. A critical avenue for future investigation is the integration of the TyG index into existing clinical risk prediction models for both CKD progression and cardiovascular disease. Studies should assess whether adding the TyG index, after adjusting for traditional confounders, can improve risk stratification, reclassification, and ultimately guide more aggressive preventive strategies in high-risk populations.

In practice, clinicians could consider using the TyG index as an initial, low-cost screening tool to identify individuals at potential risk for CKD, particularly in primary care or resource-limited settings. An elevated TyG index, especially when sustained over time, should prompt a comprehensive assessment of metabolic health, closer monitoring of renal function (eGFR and albuminuria), and aggressive management of modifiable risk factors such as dyslipidemia, hyperglycemia, and hypertension. Ultimately, advancing the standardization and clinical application of the TyG index will be crucial for optimizing CKD prevention and management, enabling earlier interventions and personalized therapies, and ultimately reducing the global burden of the disease.
